# Knowledge, attitudes, and practices of health care professionals regarding dengue fever: need for training and provision of diagnostic equipment in Togo in 2022, a cross-sectional study

**DOI:** 10.3389/fpubh.2024.1375773

**Published:** 2024-06-10

**Authors:** Roméo Mèdéssè Togan, Amadou Ibra Diallo, Wendpouiré Ida Carine Zida-Compaoré, Mouhamadou Faly Ba, Arnold Junior Sadio, Rodion Yao Konu, Akila Wimima Bakoubayi, Martin Kouame Tchankoni, Gatibe Yendu-Suglpak Gnatou, Fifonsi Adjidossi Gbeasor-Komlanvi, Fatoumata Binetou Diongue, Jean Augustin Diégane Tine, Adama Faye, Didier Koumavi Ekouévi

**Affiliations:** ^1^Department of Public Health, Faculty of Health Sciences, University of Lomé, Lomé, Togo; ^2^African Centre for Research in Epidemiology and Public Health (ACREPH), Lomé, Togo; ^3^Institute of Health and Development (ISED), Cheikh Anta Diop University, Dakar, Senegal; ^4^Training and Research Centre in Public Health, University of Lomé, Lomé, Togo; ^5^Unit 1219 - Global Health in Global South (GHiGS), Bordeaux Population Health Research Center (BPH), French National Health and Medical Research Institute (Inserm), French Research Institute for Development (IRD), University of Bordeaux, Bordeaux, France

**Keywords:** dengue, knowledge, attitudes, practices, health professional, Togo

## Abstract

**Background:**

Health statistics on dengue are virtually non-existent, despite the fact that the virus is circulating in Togo. This study aimed to assess the knowledge, attitudes, and practices (KAP) of health professionals in the Kara health region.

**Methods:**

A cross-sectional study was conducted from March to June 2022 among healthcare professionals who had worked in the Kara region of northern Togo were selected using an exhaustive recruitment method. Data were collected by trained resident doctors with a face-to-face interview using a standardized, pretested questionnaire based on the WHO 2009 dengue guide. Three multivariate regression models were utilized to investigate factors associated with knowledge, attitudes and, and practices.

**Results:**

A total of 464 respondents (37.1% female), median age 35 years, interquartile range (29–43 years) were included. Only (3.0%) of the participants had received training on dengue fever diagnosis, treatment and prevention in the last 3 years, and 10.3% had dengue rapid diagnostic tests available at their hospital. Half of the respondents (49.1%) had good knowledge of dengue fever, compared with 30.0% who had positive attitudes. Of a total of 256 professionals who had encountered a case of dengue fever in their practice, only 24 (9.4%) had appropriate practices for diagnosing and treating dengue fever. In multivariate analysis, the healthcare professionals who had taken part in ongoing training on dengue fever were more likely to have adequate dengue diagnosis and treatment practice aOR = 8.1; CI 95% = [1.7–36.0].

**Conclusion:**

Strengthening healthcare professionals' dengue-related skills through ongoing training and the provision of dengue diagnostic tests could help improve early detection practices and management of dengue fever in Togo.

## Introduction

Dengue fever, a viral infection transmitted by Aedes aegypti or Aedes Albopictus mosquito bites, is categorized among the notifiable neglected tropical diseases (1–3). It is part of the group of neglected tropical diseases that are notifiable (4, 5). Presently, it is endemic in numerous tropical and subtropical regions (6). Recent models estimate a significant rise in the global incidence of dengue fever, from 505,430 cases in 2000 to 5.2 million in 2019, with over half of the world's population being at risk in 2023 (7, 8). Currently, there is no specific treatment for dengue fever (9). Early diagnosis and treatment can reduce the mortality rate of DF patients from 20% to 1% or less (10). The role of healthcare professionals in implementing prevention, early detection, and early treatment strategies is crucial (11). Their knowledge, attitudes, and practices significantly impact the success of dengue prevention and control measures (12, 13). Studies have shown that enhancing healthcare professionals' understanding and practices of dengue is vital for early diagnosis and effective management, reducing complications, morbidity, and mortality (4, 14, 15). Most of these studies were conducted in Southeast Asian nations like Vietnam, Nepal, Thailand, and India (16–19).

In Togo, limited data exists on healthcare professionals' knowledge, attitudes, and practices (KAP) of healthcare professionals. A survey carried out in Lomé in 2020 revealed a low level of knowledge about dengue fever among healthcare professionals (20). Kara, Togo's second-largest cosmopolitan city, is near Burkina Faso, where dengue epidemics have occurred, raising concerns about disease spread (21–24). A 2022 study of 659 patients in 13 health facilities in the Kara region reported a dengue AgNS1 prevalence of 0.2% (95% CI: [0.1–0.3]). Similarly, the prevalence of IgM and IgG antibodies to dengue was 1.5% (IC95%: [0.8–2.8]) and 15.8% (IC95%: [13.2–18.8]) respectively (25). Dengue fever is therefore present in the Kara region. However, this potentially epidemic disease is under-reported and under-diagnosed throughout Togo because it is often confused with malaria.

In this context, it is vital to assess the CAPs for dengue among health professionals in the Kara region in order to identify gaps and contributing factors, an understanding of which would help in the development of strategies aimed at improving knowledge, changing attitudes and promoting appropriate practices. This study aimed to evaluate the knowledge, attitudes, and practices of healthcare professionals in the Kara health region, focusing on identifying associated factors.

## Methods

### Study area

**Figure 1:**
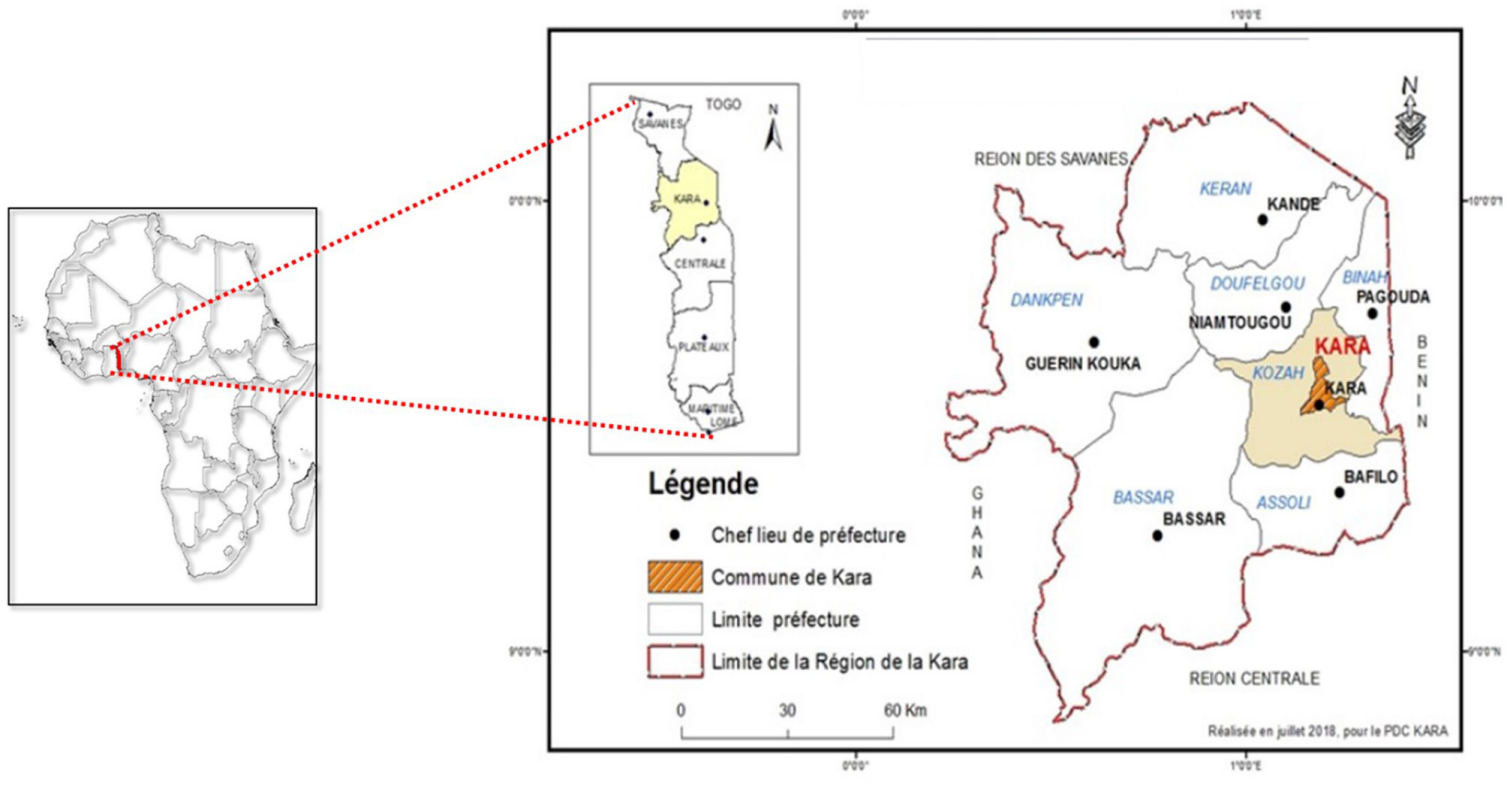
Map of the Kara health region (source Ministry of Health, Togo 2024).

The Kara region, a pivotal area in northern Togo, spans between latitudes 9°20′ and 10°05′ North and longitudes 0°55′ and 1°25′ East (Figure 1). It encompasses an area of 11,738 km^2^, accounting for nearly 21% of the country's total land area. The region's economy primarily revolves around agriculture, livestock breeding, and fishing, complemented by a robust informal sector. Two major rivers traverse the region, supporting a rich variety of vegetation. Furthermore, Kara is notable for its considerable trade and human interactions with neighboring countries, particularly Burkina Faso, which has recently confronted several dengue outbreaks (26). These factors collectively heighten the risk for the introduction and propagation of Aedes mosquito vectors in the Kara region.

### Study design

From March to June 2022 a cross-sectional study was conducted to assess KAP and associated factors related to dengue fever among health professionals in the Kara region.

### Sampling method and participant recruitment

Care professionals were recruited in an exhaustive recruitment process. All healthcare professionals working in the health facilities of the Kara region involved in the diagnosis, treatment and management of cases of dengue fever, who had given their written consent and who were present during the data collection period were included. Healthcare professionals who were absent or on sick leave during the data collection period and those who refused to participate were excluded from the study. Based on the last study carried out in 2020 on the same subject which reported a proportion of good knowledge of 47.1% in Lomé of Togo, we assume a level of knowledge of 50 % on dengue, a margin of error of 5% and a confidence interval of 95%, the sample size was initially calculated as 384 (20).

### Data collection

Face-to-face interviews were conducted using a structured questionnaire including questions on professional socio-demographic characteristics and work environment, knowledge, attitudes, and diagnostic and treatment practices regarding dengue fever. The questionnaire, based on the 2009 WHO guidelines and a review of the literature on dengue fever, was pre-tested and validated before use (8, 19, 20, 27–30) (Supplementary File 1). All the researchers were residents training as public health specialists and medical students and had been trained by experts to ensure the quality of data. Each interview lasted between 10 and 15 min. Appointments were made with the heads of the health establishments. The interviewers made daily visits to the designated healthcare establishments to administer the questionnaire to available staff. Staff who were unavailable or absent during the first visit were rescheduled during the week. Those who missed three appointments were considered to have refused to take part in the study.

### Measurements and instruments

The information collected is as follows:

- **Socio-demographic and professional characteristics**

The collected data included details such as age, sex, marital status, occupation, years of professional experience, recent participation in dengue training (within the last 3 years), primary sources of dengue information, and the availability of rapid diagnostic tests for dengue.

- **Knowledge of dengue**

We assessed participants' knowledge of DF through 10 questions covering aspects such as the distribution of dengue in Togo, the causal agent, modes of transmission, factors favoring transmission, incubation period, clinical symptoms, differential diagnoses and prevention strategies. Each correct answer was worth 1 point. The knowledge score ranged from 0 to 10 points.

- **Attitudes toward dengue fever**

Four questions were asked to assess healthcare professionals' attitudes toward dengue fever, in particular their ability to recognize a suspected case, to prevent and diagnose dengue fever in the workplace, and to recognize people at risk of dengue infection. These questions offered three possible answers: “strongly agree” and “agree”, both scored 1, and “disagree”, scored 0. As a result, the total possible attitude score ranged from 0 to 4.

- **Dengue diagnosis and treatment practices**

The assessment of practices for diagnosing, treating and preventing dengue fever was limited to participants who had already experienced a case of dengue fever. Each correct answer was worth one point, giving a score from 0 to 3.

Participants who obtained a score ≥60% for knowledge, attitudes and practices were considered to have respectively good knowledge, positive attitudes and appropriate practices for treating dengue (25, 26).

### Data analysis

The data analysis was performed using R© version 4.3.3 software. Quantitative variables were characterized using medians and interquartile ranges, while qualitative variables were described through absolute and relative frequencies. Univariable and multivariable logistic regressions were performed to assess the factors associated with knowledge, attitudes and practices regarding dengue fever among healthcare professionals. In the model building, characteristics that had a *p* < 0.25 in univariable analysis were considered for the full multivariable models, which were subsequently finalized using a stepwise backward elimination approach (p < 0.05). This procedure allowed the estimation of adjusted odds ratios (aORs) with 95% confidence intervals.

## Results

### Participation rate

**Figure 2:**
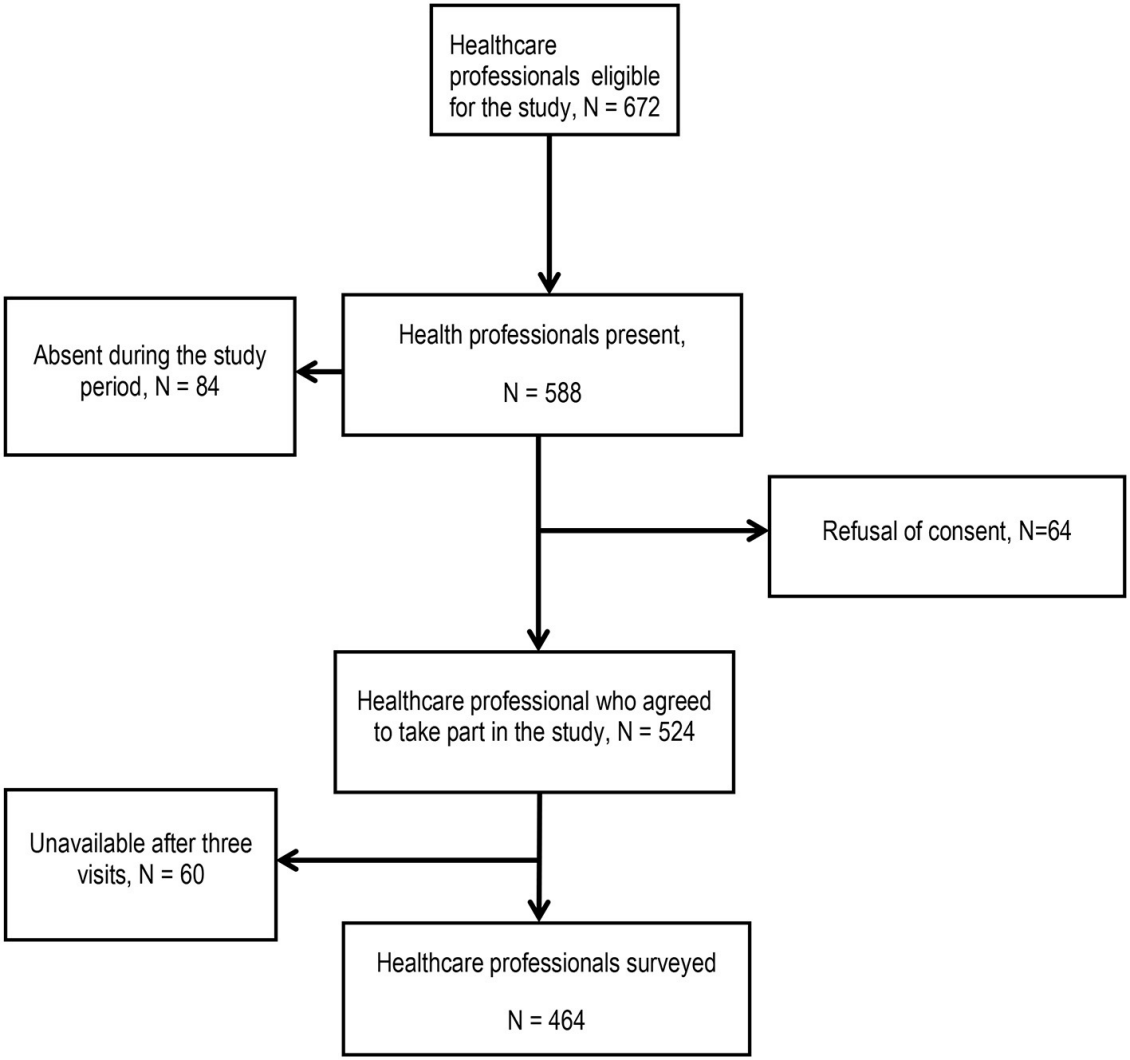
Participant flow chart.

Initially, a total of 672 healthcare professionals were targeted for exhaustive recruitment. However, during the study period, 84 of them were absent at the time of our visits for reasons of leave, on-call duty or illness, 64 refused to give their consent to participate and 60 people were not available after three visits, leaving 464 healthcare professionals representing a participation rate of 69.0%. Figure 2 shows the flow chart of patients enrolled in the study.

### Socio-demographic characteristics and professional environment

A total of 464 healthcare professionals (HCPs) with a median age of 35 years (interquartile range (IQR) 29–43 years) were questioned, 37.1% of whom were women. The majority had attained a higher level of education (72.6%), resided in the Kozah district (53.4%) and were paramedics (65.5%). Median professional experience was 8 years (IIQ: 4–14 years). The main sources of information about dengue fever were initial training (34.5%) and workplace resources (23.5%). Only 3.0% (n = 14) had received dengue training in the last 3 years. In addition, 10.3% (n = 48) indicated that their health center had dengue diagnostic equipment (Table 1).

**Table 1:** Sociodemographic characteristics and organizational environment (*N* = 464).

	**Total** ***N*** = **464**	
**Features**	**Absolute frequency (n)**	**Relative frequency (%)**
**Age range (in years)**		
Under 35	224	48.3
35 years and over	240	51.7
**Gender**		
Male	292	62.9
Female	172	37.1
**Marital status**		
In a relationship with	288	62.1
Single	176	37.9
**Higher education level**		
Yes	347	74.8
No	117	25.2
**Professional category**		
Medical staff	31	6.7
Paramedical staff	304	65.5
Hospital support staff	129	27.8
**Health district**		
Kozah	248	53.4
Assoli	30	6.5
Binah	38	8.2
Keran	71	15.3
Doufelgou	30	6.5
Bassar	23	5
Dakpen	24	5.2
**Year of professional experience**		
<10 years old	273	58.8
10 years and over	191	41.2
**Participation in training on dengue**		
Yes	14	3.0
No	450	97.0
**Availability of dengue diagnostic materials**		
Yes	48	10.3
No	416	89.7
**Sources of information on dengue**		
Initial training	160	34.5
At my place of exercise	109	23.5
On the radio or television	105	22.6
On the Internet	83	17.9
During my initial training	45	9.7

### Knowledge of dengue fever

A significant proportion of participants (70.5%) were aware of dengue fever. Regarding specific knowledge, 40.7% were familiar with the infectious agent of dengue, and 43.8% knew its incubation period. Additionally, 51.3% recognized the clinical signs, 62.1% knew the vector, and 69.6% were aware of preventive methods for dengue fever. The median knowledge score for dengue was 5.0 with an interquartile range of 2.5–7.5. Almost half of the participants, 49.1%, met the criteria for good knowledge of dengue, as defined by the 60% threshold (Table 2).

**Table 2:** Description of knowledge about dengue fever among participants (*N* = 464).

**Knowledge about dengue**	**Absolute frequency (*N*)**	**Relative frequency (%)**
**Participants who heard about dengue in Togo**		
Yes	327	70.5
No	137	29.5
**Knowledge of the distribution of dengue in Togo**		
Correct answer	72	15.5
Incorrect answer	392	84.5
**Dengue is a contagious disease**		
Correct answer	109	23.5
Incorrect answer	355	76.5
**Infectious agent responsible for dengue**		
Correct answer	189	40.7
Incorrect answer	275	59.3
**Dengue notifiable disease**		
Correct answer	270	58.2
Incorrect answer	194	41.8
**Average incubation period**		
Correct answer	203	43.8
Incorrect answer	261	56.2
**Clinical signs of dengue**		
Correct answer	238	51.3
Incorrect answer	226	48.7
**Direct cause of dengue**		
Correct answer	288	62.1
Incorrect answer	176	37.9
**Knowledge of the differential diagnosis of dengue**		
Correct answer	133	28.7
Incorrect answer	331	71.3
**Knowledge of preventive measures for dengue fever**		
Correct answer	323	69.6
Incorrect answer	141	30.4
**Median knowledge score [IIQ]**	5.0 [2.5–7.5]	
**Knowledge compared to the 60% threshold**		
Good knowledge	228	49.1
Low knowledge	236	50.9

### Attitudes toward dengue fever

Regarding attitudes, 59.3% of participants strongly agreed they could recognize a suspected dengue case, 71.8% felt confident in implementing preventive measures, and 44.2% believed they could identify vulnerable targets for dengue. Conversely, only 55 participants (11.9%) expressed confidence in their ability to diagnose dengue fever. The median attitude score among respondents was 2.0, ranging from 0 to 4, with an interquartile range of 1.0–3.0. Overall, 30% of the participants were categorized as having a positive attitude toward dengue management, based on the 60% threshold for positive attitudes (Table 3).

**Table 3:** Distribution of respondents according to attitudes toward dengue (*N* = 464).

**Attitude toward dengue**	**Absolute frequency**	**Relative frequency**
	**(** ***N*** **)**	**(%)**
**Perception of dengue diagnosis in your health facility**		
Disagree	383	82.5
Agree	26	5.6
Strongly agree	55	11.9
**Perception on vulnerable targets of dengue infection**		
Disagree	173	37.3
Agree	86	18.5
Strongly agree	205	44.2
**Perception on the recognition of a suspected case of dengue**		
Disagree	113	24.4
Agree	76	16.4
Strongly agree	275	59.3
**Perception on measures to prevent dengue**		
Disagree	61	13.1
Agree	70	15.1
Strongly agree	333	71.8
**Median dengue attitude score [IIQ]**	2.0 [1.0–3.0]	
**Attitude toward the 60% threshold**		
Positive	139	30.0
Negative	325	70.0

### Dengue-related diagnosis and treatment practices

In this study, 55.2% (256 participants) reported having encountered a suspected case of dengue fever. Among these, 64.1% (164 participants) demonstrated correct practices in diagnosing dengue, utilizing rapid diagnostic tests or serology. Furthermore, 9.4% (24 participants) had successfully managed a confirmed dengue case. The median score for dengue-related practices was 1.0, with an interquartile range (IQR) of 0.0 to 1.0, and scores ranging from a minimum of 0 to a maximum of 3. Only 9.4% (24 participants) were considered to have adequate diagnostic and treatment practices according to the 60% threshold (Table 4).

**Table 4:** Distribution of respondents according to dengue practices (*N* = 256).

**Dengue fever practices**	**Absolute frequency**	**Relative frequency**
	***N***	**(%)**
**Practices related to dengue diagnosis**		
Correct	164	64.1
Incorrect	92	35.9
**Practices for handling a suspected case**		
Correct	13	5.1
Incorrect	243	94.9
**Treatment of a confirmed case of dengue**		
Correct	24	9.4
Incorrect	232	90.6
**Dengue practice score [IIQ]**	1.0 [0.0–1.0]	
**Practice related to dengue**		
Adequate	24	9.4
Inadequate	232	90.6

### Factors associated with knowledge, attitude, and diagnosis and treatment practices of dengue fever

Multivariate logistic regression analysis revealed several factors significantly associated with good knowledge of dengue. Male healthcare professionals were more likely to possess good knowledge compared to females (aOR = 1.94; CI 95% = [1.26–3.00]). Similarly, university-educated healthcare workers were more likely to have good knowledge than others (aOR = 1.92; CI 95% = [1.14–3.26]). Coupled healthcare professionals also had good knowledge of dengue (aOR = 2.18; CI 95% = [1.38–3] 47) compared to single ones; paramedics and medical staff were more likely to have good knowledge of dengue with aOR = 1.88 [1.13–3.16] and aOR = 6.53; CI 95% = [2.32–21.6] respectively. Furthermore, participants with more than 10 years of experience were 2.3 times less likely to have good knowledge than respondents with <10 years of professional experience. In addition, the availability of dengue diagnostic equipment was associated with good dengue-related knowledge aOR = 2.19; CI 95% [1.13–4.39].

About factors linked to positive attitudes, the availability of dengue diagnostic equipment was significantly associated with a positive attitude toward dengue (aOR = 2.94; IC 95% = [1.46–5.98]). medical staff had a positive attitude compared to other professional categories (aOR = 4.81; IC 95% = [1.86–12.8]). In addition, staff in the six other districts compared with those in the Kozah district had a better positive attitude to dengue fever (aOR = 2.8; CI 95% = [1.751–4.55]). Finally, healthcare professionals with good knowledge of dengue fever were more likely to have a positive attitude toward dengue compared with those with poor knowledge (aOR = 6.06; CI 95% = [3.71–10.2]).

In terms of factors associated with adequate dengue diagnosis and treatment practices, healthcare staff who had received dengue training in the last 3 years were more likely to have adequate dengue practice (aOR = 8.14; CI 95% = [1.72–36.0]). Information on factors associated with dengue CAP among health professionals in the Kara region is presented in Table 5.

**Table 5:** Factors associated with knowledge, attitudes and practices related to dengue.

**Features**	**Good knowledge multivariate model**			**Multivariate positive attitude model**			**Adequate practical multivariate model**		
	**ORa**	**95% CI**	***p*** **-value**	**ORa**	**95% CI**	***p*** **-value**	**ORa**	**95% CI**	***p*** **-value**
**Sex**									
Female	1	—							
Male	1.94	1.26–3.00	**0.003**						
**Higher education level**									
No	1	—							
Yes	1.92	1.14–3.26	**0.015**						
**Marital status**									
Single	1	—							
In a relationship with	2.18	1.38–3.47	**<0.001**						
**Professional category**									
Support hospitalist	1	—		1	—		1	—	
Paramedical	1.88	1.13–3.16	**0.015**	1.64	0.93–2.96	0.092	0.69	0.23–2.44	0.540
Medical	6.53	2.32–21.6	**<0.001**	4.81	1.86–12.8	**0.001**	3.45	0.93–13.6	0.073
**Year range of professional experience (year)**									
<10 years old	1	—							
10 years and over	0.43	0.27–0.67	**<0.001**						
**Participation in training on dengue**									
No							1	—	
Yes							8.14	1.72–36.0	**0.006**
**Availability of equipment dengue diagnosis**									
No	1	—		1	—		1	—	
Yes	2.19	1.13–4.39	**0.022**	2.94	1.46–5.98	**0.003**	1.36	0.33–4.41	0.637
**Knowledge about dengue**									
Weak				1	—		1	—	
Good				6.06	3.71–10.2	**<0.001**	1.08	0.37–3.73	0.893
**Attitudes toward dengue**									
Negative							1	—	
Positive							0.83	0.32–2.06	0.683

ORa, adjusted odds ratio; CI, Confidence interval. The bold values represent statistically significant results (*p* < 0.05).

## Discussion

### Good knowledge of dengue among healthcare workers in the Kara region and associated factors

This 2022 study in Kara, northern Togo, found that 49.1% of healthcare professionals had good knowledge of dengue fever, a figure similar to the 47.1% reported by Zida-compaoré et al. in 2021 in Lomé, southern Togo (20). These results suggest a relatively low level of dengue knowledge among Togolese healthcare workers, lower than figures reported elsewhere. For instance, Yusuf et al. in Ethiopia (2019) found 59.6% good knowledge (29), while Koonisetty in Turkey and Oche et al. in Nigeria (2021) reported 69.0% and 87.8%, respectively (19, 28). The limited knowledge in Togo may be attributed to factors like insufficient ongoing training for healthcare personnel and inadequate dengue awareness among both healthcare staff and the public community. Additionally, the infrequent encounter with dengue cases in Togo, as opposed to recent epidemics in other countries that have prompted increased learning, might contribute to this knowledge gap (31–33). The presence of dengue diagnostic equipment was significantly linked to better knowledge (aOR = 2.19; CI 95% [1.13–4.39]). However, only 10.3% of surveyed professionals reported having access to rapid dengue diagnostics. This scarcity could contribute to the underreporting of dengue, which has epidemic potential in Kara, as seen in national health statistics (34). Similar patterns of underreporting were observed in many non-endemic countries, possibly due to the low prevalence of reported cases in Africa despite conducive climatic conditions for dengue transmission (2, 35). Therefore, enhancing diagnostic access and affordability is crucial for the early detection of dengue in Togo.

### Positive attitudes toward dengue fever among healthcare workers in the Kara region

In our study, only 30.0% of surveyed healthcare workers exhibited a positive attitude toward dengue fever, possibly reflecting the low proportion of good knowledge about dengue among participants. This correlation mirrors findings from a 2020 Malaysian study, where 46.8% of healthcare personnel had a positive attitude toward dengue, paralleling the 50.7% with good knowledge (36). Similarly, a 2019 Ethiopian study reported a 48% rate of positive attitudes among healthcare workers (29), whereas a 2021 Nigerian study by Oche et al. found a significantly higher rate of 93.2% positive attitudes, aligned with 87.8% good knowledge (28). These comparisons underscore the link between knowledge of dengue and positive attitudes toward its management among healthcare providers. Regarding factors influencing positive attitudes toward dengue fever, our multivariate analysis indicated that medical staff were more likely to exhibit positive attitudes than other professional groups (aOR = 4.81; CI 95% = [1.86–12.8]), a finding consistent with studies by Zida-Compaor et al. (20) in Togo, Oche et al. (28) in Nigeria, and Mohammed Yusuf and Abdurashid Ibrahim (29) in Ethiopia. This trend could be attributed to the frontline role of medical staff in managing dengue cases, fostering more positive attitudes toward the condition compared to other healthcare professionals. Moreover, the presence of dengue diagnostic tools was notably correlated with a favorable attitude, as indicated by an adjusted odds ratio (aOR) of 2.94 and a 95% confidence interval (CI) of [1.46–5.98]. This finding aligns with the work of Wong et al. (37) in Malaysia, who documented an aOR of 3.09 [1.27–7.5]. Such evidence highlights the critical role of ready access to rapid diagnostic tests at health facilities in fostering positive attitudes among healthcare workers toward dengue. Furthermore, healthcare professionals with comprehensive knowledge of dengue fever were significantly more inclined to demonstrate a positive attitude compared to their less-informed peers, with an aOR of 6.06; CI 95% = [3.71–10.2], affirming consistency with previous literature (19, 28, 29).

### Appropriate dengue-related diagnosis and treatment practices among healthcare workers in the Kara region

In our investigation, a mere 9.4% of healthcare professionals with experience handling dengue cases demonstrated sufficient dengue-related practices, a strikingly low figure relative to those reported in existing research. For comparison, Yusuf and colleagues identified that in Ethiopia in 2019, 48% of surveyed healthcare workers exhibited appropriate dengue-related practices based on a 60% cut-off (29). Similarly, using a 70% cut-off, Nikookar et al. reported that in Iran in 2023, an impressive 73.0% of participants had good practices (38). The scant proportion of adequate dengue practices in our study could primarily be attributed to the reported unavailability of dengue diagnostic equipment within the participants' health region. Furthermore, the observed deficiency in ongoing training, coupled with the lower scores in knowledge and attitudes, may contribute to the limited extent of adequate practices. Notably, healthcare professionals who had received training on dengue in the past 3 years were significantly more likely to engage in adequate dengue practices, as evidenced by an adjusted odds ratio (aOR) of 8.14 and a 95% confidence interval (CI) of [1.72–36.0]. The implications of our results highlight the critical need for targeted interventions to ensure the availability of dengue diagnostic equipment in health facilities in the Kara region. Secondly, it is essential to invest in comprehensive and ongoing training programs on dengue management. In addition, the promotion of multidisciplinary approaches that involve coordinated efforts in surveillance, vector control and community engagement can help mitigate the impact of dengue in the Kara region. Health professionals can play a key role in disseminating accurate information and promoting preventive behavior about dengue among the population (39).

### Limitations

Some potential limitations that could affect the accuracy and reliability of the results were identified and controlled before the study was carried out. These were selection bias, social desirability bias and statistical analysis limitation. With regard to selection bias, the study attempted to minimize these threats by including all health professionals working in all health facilities in the Kara region with exhaustive recruitment. Concerning social desirability bias, this was possible due to the face-to-face administration of the questionnaire. Participants might feel obliged to answer in an acceptable way or in accordance with the majority opinion in order to please, rather than answering honestly and objectively. To avoid this, the interviewers were trained to administer the questionnaire in order to reassure participants that their anonymity, confidentiality and freedom of expression would be preserved in accordance with the ethical and deontological rules of research. Similarly, the questionnaire was administered in an office with only the participant facing the interviewer, to avoid the influence of colleagues and limit social desirability bias. For the statistical analysis, we set a significance level of 0.05 based on the literature review and given that our study was exploratory in nature. We did not make any corrections in order to guarantee comparability with the data found in the literature, which used the same 0.05 threshold (16, 20, 28, 38). Despite these limitations, most of which were anticipated and corrected, our results were consistent with those of Ida et al. in Lomé and can therefore be generalized to Togo (20).

## Conclusion

In conclusion, our study highlighted alarming gaps in dengue diagnosis and treatment practices among health professionals in the Kara region. This finding highlights the urgent need for targeted interventions to address the challenges of improving access to diagnostic equipment, strengthening training programs and improving public awareness. Our results also pave the way for other avenues of research into dengue, particularly within the community, with a view to advancing dengue prevention and control. This includes developing robust surveillance systems to monitor the incidence of dengue and identify early warning signs of dengue epidemics. In addition, efforts to monitor vectors need to be stepped up in order to track mosquito populations and the dynamics of disease transmission. Finally, socio-behavioral research is needed to understand the perceptions, knowledge and practices of communities in terms of dengue prevention and control in the Kara region.

## Data availability statement

The raw data supporting the conclusions of this article will be made available by the authors, without undue reservation.

## Ethics statement

The studies involving humans were approved by Bioethics Committee for Health Research. The studies were conducted in accordance with the local legislation and institutional requirements. The participants provided their written informed consent to participate in this study.

## Author contributions

RT: Conceptualization, Data curation, Formal analysis, Investigation, Methodology, Writing – original draft, Writing – review & editing. AD: Methodology, Supervision, Writing – review & editing. WZ-C: Investigation, Methodology, Validation, Writing – review & editing. MB: Methodology, Supervision, Visualization, Writing – review & editing. AS: Data curation, Formal analysis, Investigation, Methodology, Validation, Writing – review & editing. RK: Investigation, Methodology, Writing – review & editing. AB: Investigation, Methodology, Writing – review & editing. MT: Data curation, Formal analysis, Supervision, Visualization, Writing – review & editing. GG: Investigation, Validation, Writing – review & editing. FG-K: Conceptualization, Project administration, Supervision, Validation, Writing – review & editing. FD: Supervision, Validation, Writing – review & editing. JT: Conceptualization, Supervision, Validation, Writing – review & editing. AF: Conceptualization, Methodology, Resources, Supervision, Validation, Visualization, Writing – review & editing. DE: Conceptualization, Methodology, Resources, Supervision, Validation, Writing – review & editing.
