# Supplementary material for: Knowledge, attitudes, and practices of health care professionals regarding dengue fever: need for training and provision of diagnostic equipment in Togo in 2022, a cross-sectional study
Source: Front Public Health. 2024 Jun 10;12:1375773. doi: 10.3389/fpubh.2024.1375773 (PMC11194359; doi:10.3389/fpubh.2024.1375773)
Supplement: Supplementary file 1 [file Data_Sheet_1.pdf]

DENGUE KNOWLEDGE, ATTITUDES AND PRACTICES (KAP) SURVEY QUESTIONNAIRE FOR HEALTHCARE WORKERS IN THE KARA REGION

Information

Hello, the aim of this study is to assess the knowledge, attitudes and practices of healthcare staff in the Kara region regarding dengue fever, in order to improve the management of patients presenting with fever. This questionnaire is completely anonymous and will take about 5 minutes to complete.

Consent

Do you agree to take part in the study? □ Yes□ No

If No, end of questionnaire.

If Yes, go to section I after entering the date and respondent ID.

Survey date: ______/______/_________ ID : _______________________

Section I Socio-demographic, professional and work environment characteristics	
Variables	**Terms and conditions**
Age	|___| |___| Number in years
Gender	0= Female; 1= Male
Marital status	1= Married; 0 = Unmarried (Single, Divorced and Widowed)
Professional qualifications	1= Doctor; 2= Nurse; 3= Medical assistant<br>4= Midwife; 5= Laboratory technician<br>6= Orderly ;
Professional category	1= Medical; 2= Paramedical; 3= Hospital support
Education level	1 = Higher 0= (Primary; Secondary; Out of school)
Health District	1= Keran; 2 =Bassar; 3=Dakpen; 4=Kozah; 5= Binah;<br>6= Doufelgou; 7= Assoli;
Level of care in the health pyramid	1 = Peripheral level 2= Intermediate level<br>3 = Tertiary level
Number of years of service since appointment	|___| |___| Number in years
Source of information on dengue fever	1=During initial training, 2=At my practice,3=During continuing training<br>4= Radio/TV, 5= Internet
Have you ever taken part in a dengue training course?	1=Yes, 0=No
Do you have the necessary equipment at your facility to diagnose dengue fever?	1=Yes, 0=No
**Section II: Knowledge about dengue fever**	
Have you ever heard of dengue fever in Togo?	**1=Yes**, 0=No

Knowledge of dengue distribution in Togo	**1= Lomé-commune, 2= Maritime, 3=Plateaux, 4=Centrale, 5= Kara, 6= Savanes**<br>**NB: Correct answer if at least three regions ticked**
Is dengue a contagious disease?	**1=No,** 0=Yes
What type of infectious agent is responsible for cases of dengue fever?	**1=Viral**, 2=Bacterial, 3=Parasitic, 4=Mycosic, 5=Don't know
Is dengue a notifiable disease?	**1=Yes**, 0=No
What is the average incubation period for dengue infection?	**1= less than 7 days 2= 7 to 14 days** 3= more than 14 days
What are the clinical signs of dengue fever?	**1= Headache, 2= Fever, 3=Rash, 4=Vomiting, 5= Joint pain, 6= Cough, 7= Bleeding, 8= Sore throat, 9= Asthenia, 10= Conjunctivitis** 11= Other ________<br>**NB: Correct answer if at least three clinical signs listed**
What is the direct cause of dengue fever?	1= Poor diet, 2= Dirty environment**, 3= Bites from Aedes mosquitoes, 4=** Polluted water, 5= Contact with blood products,<br>6= Lack of personal hygiene, 7= Don't know
What other diseases do you think of when faced with a suspected case of dengue?	**1=Malaria, 2=COVID-19, 3=Yellow fever 4=Ebola, 5=Lassa fever, 6=Typhoid fever** 7=Don't know<br>**NB: Correct answer if at least three diagnoses evoked**
What can be done to prevent dengue fever?	1=Healthy diet,**2=Use of LLINs, 3=Use of repellents, 4=Sanitation of living environment, 5=Elimination of stagnant water**<br>6=Condom use 7=Good hand hygiene **8=Insecticide use**<br>**NB: Correct answer if at least three of the means listed in bold are used.**

**Section III: Attitudes towards dengue fever**	
Can you recognize a suspected case of dengue fever?	**1=Strongly agree, 2 =Agree**,3 = Disagree
Do you think dengue fever can be prevented?	**1=Strongly agree, 2 =Agree**, 3 = Disagree
Do you think you can diagnose dengue fever at your facility?	**1=Strongly agree, 2 =Agree,** 3 = Disagree
Do you think that children, pregnant women, the elderly and people living with HIV are at greater risk of catching dengue fever?	**1=Strongly agree, 2=Agree,** 3 = Disagree
**Section IV: Dengue-related practices**	
Have you ever been confronted with a suspected case of dengue fever?	**1=Yes,** 0=No

When faced with a suspected case of dengue fever, what diagnostic tools will you use to make the diagnosis?	**1=TDR, 2=Serology**, 3=Culture<br>4= Don't know
Have you ever treated a case of dengue fever?	**1=Yes**, 0=No
What kind of treatment do you use?	**1=Symptomatic treatment, 2=Ribavirin**,<br>**3=Isolation**<br>**If at least 1 out of 3 is ticked = correct answer**
